# Nifuroxazide ameliorates pulmonary fibrosis by blocking myofibroblast genesis: a drug repurposing study

**DOI:** 10.1186/s12931-022-01946-6

**Published:** 2022-02-16

**Authors:** Cailing Gan, Qianyu Zhang, Hongyao Liu, Guan Wang, Liqun Wang, Yali Li, Zui Tan, Wenya Yin, Yuqin Yao, Yongmei Xie, Liang Ouyang, Luoting Yu, Tinghong Ye

**Affiliations:** 1 Sichuan University-Oxford University Huaxi Gastrointestinal Cancer Centre, State Key Laboratory of Biotherapy, West China Hospital, Sichuan University, 17# 3rd Section, Ren Min South Road, Chengdu, 610041 China; 2 Department of Nutrition and Food Hygiene, School of Public Health, West China Medical School, Sichuan University, Chengdu, 610041 China; 3 Innovation Center of Nursing Research, West China Hospital, Sichuan University, Chengdu, 610041 China; 4 Nursing Key Laboratory of Sichuan Province, Sichuan University, Chengdu, 610041 China

**Keywords:** Nifuroxazide, IPF, TGF-β1/Smad, Stat3, EMT

## Abstract

**Background:**

Idiopathic pulmonary fibrosis (IPF) is a serious interstitial lung disease with a complex pathogenesis and high mortality. The development of new drugs is time-consuming and laborious; therefore, research on the new use of old drugs can save time and clinical costs and even avoid serious side effects. Nifuroxazide (NIF) was originally used to treat diarrhoea, but more recently, it has been found to have additional pharmacological effects, such as anti-tumour effects and inhibition of inflammatory diseases related to diabetic nephropathy. However, there are no reports regarding its role in pulmonary fibrosis.

**Methods:**

The therapeutic effect of NIF on pulmonary fibrosis in vivo was measured by ELISA, hydroxyproline content, H&E and Masson staining, immunohistochemistry (IHC) and western blot. Immune cell content in lung tissue was also analysed by flow cytometry. NIF cytotoxicity was evaluated in NIH/3T3 cells, human pulmonary fibroblasts (HPFs), A549 cells and rat primary lung fibroblasts (RPLFs) using the MTT assay. Finally, an in vitro cell model created by transforming growth factor-β1 (TGF-β1) stimulation was assessed using different experiments (immunofluorescence, western blot and wound migration assay) to evaluate the effects of NIF on the activation of NIH/3T3 and HPF cells and the epithelial-mesenchymal transition (EMT) and migration of A549 cells.

**Results:**

In vivo, intraperitoneal injection of NIF relieved and reversed pulmonary fibrosis caused by bleomycin (BLM) bronchial instillation. In addition, NIF inhibited the expression of a variety of cellular inflammatory factors and immune cells. Furthermore, NIF suppressed the activation of fibroblasts and EMT of epithelial cells induced by TGF-β1. Most importantly, we used an analytical docking experiment and thermal shift assay to further verify that NIF functions in conjunction with signal transducer and activator of transcription 3 (Stat3). Moreover, NIF inhibited the TGF-β/Smad pathway in vitro and decreased the expression of phosphorylated Stat3 in vitro and in vivo.

**Conclusion:**

Taken together, we conclude that NIF inhibits and reverses pulmonary fibrosis, and these results support NIF as a viable therapeutic option for IPF treatment.

**Graphic Abstract:**

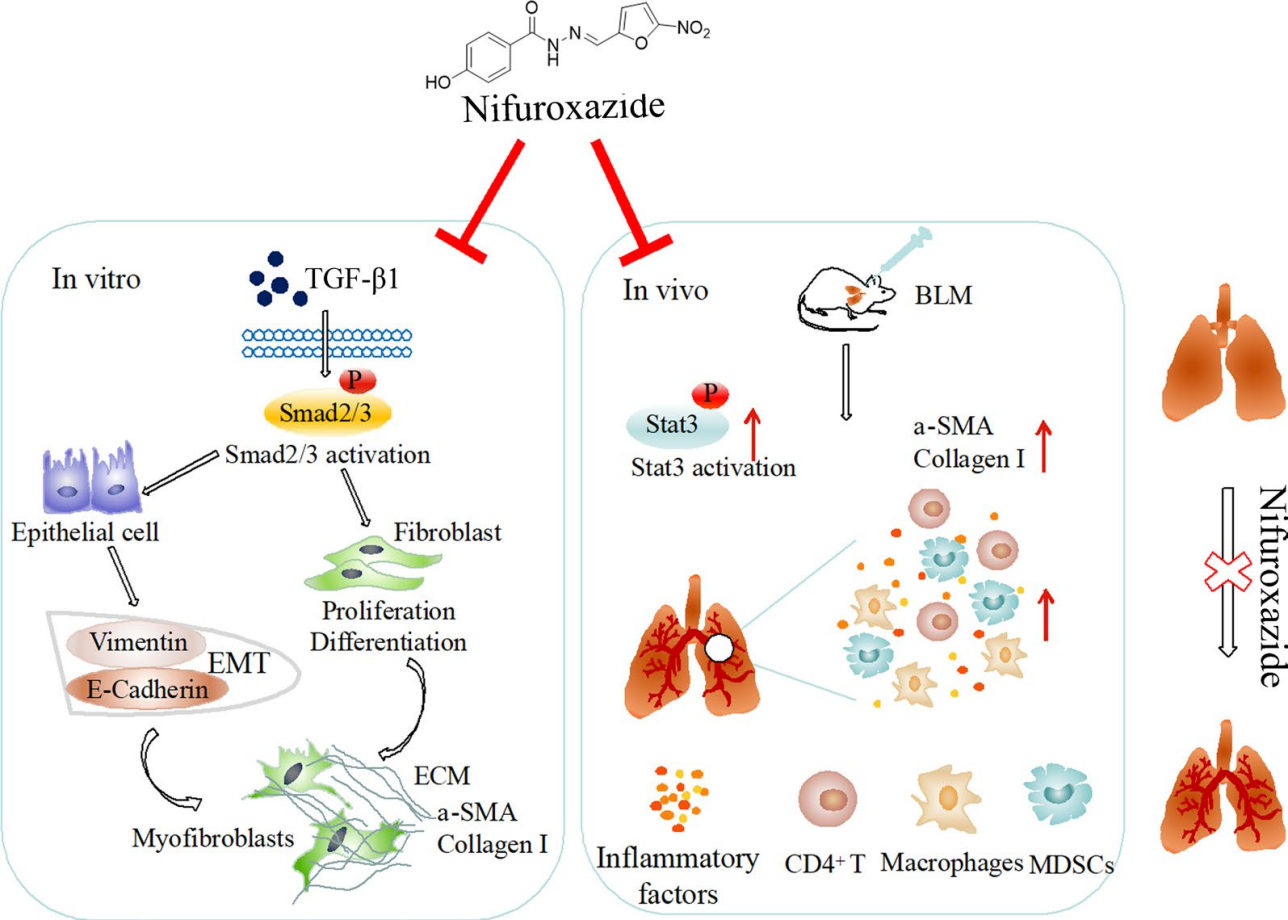

**Supplementary Information:**

The online version contains supplementary material available at 10.1186/s12931-022-01946-6.

## Background

Pulmonary fibrosis is a type of unrepairable and difficult-to-treat progressive interstitial lung disease, of which idiopathic pulmonary fibrosis (IPF) is the most severe [1]. IPF usually occurs in adults, causing dyspnoea and dry cough, and has a median survival of 2–3 years [2, 3]. Pulmonary fibrosis is caused by ageing, genetic factors, environmental factors and other factors, leading to the abnormal activation of fibroblasts into myofibroblasts and excessive proliferation, secreting large amounts of collagen and other extracellular matrix (ECM) components, thereby damaging the lung structure and rapidly reducing lung function [4, 5]. There is no standard of care for the treatment of pulmonary fibrosis, although there are currently two drugs on the market, nintedanib and pirfenidone [6]. However, nintedanib does not significantly improve the rate of forced vital capacity (FVC) ecline in patients with nonadvanced pulmonary fibrosis, and the incidence of adverse reactions is 97.2% [7]. Furthermore, pirfenidone causes several gastrointestinal side effects, nausea and other adverse reactions [8]. To date, reversing pulmonary fibrosis remains challenging. Therefore, potential drugs are urgently needed to treat pulmonary fibrosis.

The progression of pulmonary fibrosis is regulated by various inflammatory factors and chemokines and involves multiple signalling pathways. Transforming growth factor-β1 (TGF-β1) is one of the most effective inducing factors among these molecules [9]. TGF-β1 stimulates fibroblast activation and epithelial-mesenchymal transition (EMT), accelerates ECM accumulation, and aggravates lung fibrosis by interacting with TGF-β receptors [10]. In addition, signal transducer and activator of transcription 3 (Stat3), which regulates multiple cellular functions, including proliferation, migration, survival and differentiation, is another key molecule that regulates the phenotype of fibroblasts [11]. It has been reported that the Stat3 signalling pathway is activated in fibroblasts, and inhibition of the Stat3 pathway can reverse fibroblast activity [12]. Therefore, this evidence suggests that suppression of the TGF-β1 and Stat3 signalling pathways could reduce pulmonary fibrosis by inhibiting fibroblast activation and excessive ECM expression.

Given that the discovery and development of new drugs is a very long and extremely costly process, it is more attractive to repurpose “old” drug treatments because they are typically low-risk compounds that may save in development costs and time [13]. As an inhibitor of Stat3, nifuroxazide (NIF) was originally used to treat diarrhoea, but in recent years, it has been found to have effects against breast cancer, primary myeloma, hepatocarcinoma and other cancers and promotes the immune response against tumours [14–16]. In addition, NIF significantly reduces renal macrophage infiltration and fibrosis in diabetic kidney tissue [17]. However, there are no data regarding the role of NIF in IPF.

In this study, we found that NIF may regulate the TGF-β/Smad and Stat3 pathways, inhibiting the activation of fibroblasts and EMT and the migration of A549 epithelial cells, which alleviated pulmonary fibrosis. These results suggest that NIF is a potential drug for the treatment of pulmonary fibrosis.

## Methods

### Reagents and antibodies

NIF was purchased from Xiyashiji Chemical Co., Ltd. (Chengdu, Sichuan, China). For the in vitro study, a 20 mM/50 mM NIF solution was prepared in dimethyl sulfoxide (DMSO) (Sigma, St Louis, Mo), stored at − 20 ℃ and diluted in culture medium to the required concentration. When the DMSO concentration was < 1‰, the culture medium was used as the vehicle control; when the DMSO concentration was ≥ 1‰, the same concentration of DMSO was used as the vehicle control. For in vivo studies, NIF was prepared at a 5:35:60 ratio of DMSO: polyethylene glycol 400 (PEG 400) (Sigma, St. Louis, MO, USA): normal saline and administered at a dose of 0.1 mL/10 g body weight. BLM sulphate was purchased from Chengdu Synguider Technology Co., Ltd. (Chengdu, China). Collagenase Type IV was purchased from Gibco (#17104-019; Grand Island, NY, USA). TGF-β1 was purchased from Novoprotein (#CA59; Shanghai, China). Primary antibodies against β-actin (ab8226; Abcam, Cambridge, MA, USA), GAPDH (TA-08; ZSGB-BIO, Beijing, China), α-SMA (ab5694), Collagen-I (ab88147), E-Cadherin (ab76055) and Vimentin (ab20346) were purchased from Abcam (Cambridge, MA, USA), and Smad2/3/phospho-Smad2/3 (#8685; #8828) and Stat3/phospho-Stat3^TY705^ (#9139; #9145) were purchased from Cell Signaling Technology Company (MA, USA). PE-CD11b (#12-0112-82), PE-CD4 (#12-0041-82), APC-CD69 (#17-0691-82), and FITC-CD8 (#11-0081-82) were purchased from BD Biosciences (San Diego, CA, USA). PE-F4/80 (#123110), FITC-CD11b (#101206), and FITC-Gr-1 (#108406) were purchased from Biolegend (San Diego, CA, USA).

### Cell culture

A549 (human alveolar basal epithelial cells) and NIH/3T3 (mouse embryonic fibroblasts) were purchased from ATCC (Rockville, MD, USA). HPFs (human pulmonary fibroblasts) were purchased from Science Cell (San Diego, CA, USA). The three cell types were cultured in DMEM (Gibco, Grand Island, NY, USA) supplemented with 10% or 20% heat-inactivated foetal bovine serum (FBS; HyClone, Logan, UT, USA) and 1% penicillin/streptomycin (MP Biomedical LLC) in 5% CO_2_ at 37 °C.

### Molecular docking studies

The three-dimensional X-ray crystal structure of Stat3 (PDB ID: 6NJS) was downloaded from the Protein Data Bank [18]. Both the compound and protein were processed using the CHAR Mm force field [19]. Molecular docking was performed using the CDOCKER module in Accelrys Discovery Studio (version 3.5; Accelrys, San Diego, CA, USA). The molecular docking parameters were determined according to the standard values set by the software. After the docking study was completed, the platform was used to collect the docking score and analyse the docking modes.

### MTT assay

The thiazolyl blue tetrazolium bromide (MTT) (Sigma, St Louis, MO) assay was used to evaluate the viability of NIF-treated cells. Cells were cultured in 96-well plates at a density of 1000–8000 cells/well and administered NIF (0–20 μM) 24 h later. Next, after coincubation for 24, 48, and 72 h, 20 μL of 5 mg/mL MTT was added to each well, and cells were further incubated at 37 °C for 2–4 h. A Spectra MAX M5 microplate spectrophotometer (Molecular Devices, Sunnyvale, CA, USA) was used to determine the absorbance at 570 nm.

### Immunofluorescence analysis

Cells were cultured at a concentration of 10,000–20,000 cells/well in 24-well plates containing glass slides (14 mm × 14 mm). After 48 h of culture, the medium was discarded and replaced with serum-free DMEM for 6 h. Then, the medium was replaced with complete medium, and 5 ng/mL TGF-β1 was added. After 1 h, NIF (20 μM) was added and incubated for another 24 h. Next, the slides were fixed in 4% paraformaldehyde for 15 min at room temperature followed by washing 3 times with PBS and permeabilizing with 0.5% Triton X-100 (Sigma, St Louis, MO) for 20 min. After washing 3 times with PBS, cells were blocked in 5% BSA (BioFroxx) solution at room temperature for 30 min followed by incubation with primary antibodies (anti‐E‐cadherin (1:200; Abcam, Cambridge, MA, USA) and anti-α-SMA (1:200; Abcam, Cambridge, MA, USA)) overnight at 4℃. The slides were subsequently washed 3 times with PBS (5 min each time) and incubated with the secondary antibody (Cy3‐labelled goat anti‐mouse immunoglobulin G (IgG; Beyotime, Shanghai, China) at a 1:200 dilution (red) or FITC-488‐labelled goat anti‐rabbit immunoglobulin G (IgG; Beyotime, Shanghai, China) at a 1:200 dilution (green)) for 1 h at room temperature. The slides were then washed 3 times with PBS and stained with DAPI (Biosharp, Hefei, China) at room temperature for 10 min. After washing 3 times with PBS, an anti-fluorescence quencher (IgG; Beyotime, Shanghai, China) was added. Immunofluorescence was analysed under a fluorescence microscope (Zeiss LS880, Germany).

### Wound migration assay

A549 cells were inoculated into 6-well plates at a density of 10^5^ cells/well. When the cells had reached approximately 80% confluence, the medium was discarded and replaced with serum-free medium. After incubation for 6 h at 37 ℃, the cells were scratched using 200 μL pipette tips. The cells were washed with PBS to remove cellular debris and cultured in complete medium (less than 3%) containing TGF-β1 (5 ng/mL). One hour later, NIF (20 μM) was added, and images of the scratches were acquired 0 and 24 h later using a microscope (Olympus, ix73, Japan). The wound closure rate was calculated as the ratio of the cell migration area and the original wound area.

### Cell/tissue lysates and western blot

Protein detection were performed in accordance with previous reports [20]. Cells were inoculated into Petri dishes (10 cm) and then treated with the same immunofluorescence procedure as those collected after 24 h of NIF administration. Collected tissue was frozen in liquid nitrogen and ground into a powder in a mortar. Lung tissue and cultured cells were homogenized in RIPA buffer (Beyotime, Shanghai, China) supplemented with protease and phosphatase inhibitors (Selleckchem) and quantified using the Bradford method. Then, the proteins were separated on 10% SDS–PAGE gels (Chengdu Baihe Technology Co., Ltd.) and transferred onto 0.45 μM PVDF membranes (Merck Millipore, Billerica, MA, USA). After blocking in 5% (M/V) non-fat milk for 1 h, the membranes were incubated with antibodies overnight at 4 °C. Then, the membranes were incubated with goat anti‐rabbit/mouse IgG (ZSGB-BIO Co., Beijing, China) at a 1:3000 dilution for 60 min at 37 °C. Reactive bands were identified using an enhanced chemiluminescence kit (Merck Millipore, Billerica, MA, USA). Then, the images were analysed using ImageJ software (National Institute of Health, Bethesda, MD, USA).

### Thermal drift assay

These methods were performed according to those reported in the literature [21] with some modifications. Cells were cultured in the same manner described above for protein extraction. After treatment with NIF (200 μM) for 2 h, the cells were collected, PBS containing protease inhibitor was added, and the cells were adjusted to the same concentration after cell counting. The cell suspension was divided into 8 equal parts and heated at 42, 44, 46, 48, 50, 52, 54 and 56 °C for 3 min, frozen in liquid nitrogen (1 min) and re-dissolved at room temperature three times. After centrifuging the samples at 13,300 r/15 min, the final protein was obtained by removing the supernatant. Then, protein images were obtained and analysed according to the method described above.

### Isolation and culture of lung fibroblasts

Wistar rats (male; 6–8 weeks; 180–220 g) were purchased from Vital River (Beijing, China). Rats were anaesthetized using 10% chloral hydrate and then intratracheally administered BLM (5 mg/kg). After 14 days, the rats were sacrificed, and complete lung tissue was obtained by dissection. Then, the lungs of the rats were washed with Hanks solution, minced and digested in trypsin. After incubation at 37 °C for 40 min, the cell suspension was filtered through a 70 μM screen mesh. The filtrate was centrifuged at 1500 rpm/5 min and the precipitates were collected, mainly lung fibroblasts and epithelial cells. The precipitate was resuspended in the medium and centrifuged at 800 rpm for 5 min. Take the supernatant (mainly including lung fibroblasts and a few epithelial cells), discard the precipitate (mainly including epithelial cells), centrifuge the obtained supernatant at 1500 r/5 min, and then collect the sediment containing lung fibroblasts [22, 23]. The extracted precipitate containing primary lung fibroblasts were cultured in DMEM/F-12 (GIBCO, NY, USA) medium containing 10% FBS and 1% penicillin and streptomycin and streptomycin in 5% CO_2_ at 37℃. After culturing in the incubator for 40 min, discard the non-adherent cells (Lung fibroblasts adhere faster and can be separated from other cells) [24]. The adherent cells are pure lung fibroblasts and cultured in fresh culture medium. This method was also used in the subsequent passage to further purify the extracted lung fibroblasts. Lung fibroblasts were identified by morphology and immunofluorescence (Additional file 1: Fig. S1a, b) [25]. The cells were used between passages 3 and 8.

### Animal studies of fibrosis

C57BL/6 mice (6–8 weeks) were purchased from Huafukang (Beijing, China). C57BL/6 mice were anaesthetized using 10% chloral hydrate and then intratracheally administered BLM, and the sham-operated group was administered with the same volume of physiological saline.

In the preventive model, mice were intratracheally administered BLM (~ 2.5 mg/kg) while the sham-operated group was administered with the same volume of physiological saline. All bronchial drip only once at day 0. On the first day of BLM bronchial drip, all BLM mice were randomly divided into 3 groups: n = 14 (Vehicle); n = 10 (NIF 25 mg/kg); n = 14 (NIF 50 mg/kg). The sham operation group (n = 10) and the vehicle group were intraperitoneally injected with dissolvent (5:35:60 ratio of DMSO: polyethylene glycol 400: normal saline) every day for 27 days. At the same time, the NIF groups received intraperitoneal injection of low-dose NIF (25 mg/kg) or high-dose NIF (50 mg/kg) every day. Mice were sacrificed on day 28.

In the therapeutic model, mice were intratracheally administered BLM (~ 1 mg/kg) while the sham-operated group was administered with the same volume of physiological saline. All bronchial drip only once at day 0. On the first day of BLM bronchial drip, the health status of mice was observed, and the previous feeding pattern was maintained until the 13th day after modelling. On the 14th day, all BLM mice were randomly divided into 3 groups. n = 9 (Vehicle), n = 8 (25 mg/kg, 50 mg/kg NIF). The sham operation group (n = 4) and the vehicle group were began intraperitoneally injected with dissolvent (5:35:60 ratio of DMSO: polyethylene glycol 400: normal saline) every day on day 14. At the same time, the NIF groups received intraperitoneal injection of low-dose NIF (25 mg/kg) or high-dose NIF (50 mg/kg) every day. Mice were sacrificed on day 28.

On the 28th day, some mice were used for the extraction of bronchoalveolar lavage fluid (BALF), and the remaining mice were used to collect blood from the eyeballs into EP tubes without coagulant. After standing at 4℃ for almost 24 h, the blood was centrifuged at 3000*g*/10 min to obtain the serum for ELISA detection.

### Extraction of BALF

BALF was extracted according to previous methods reported in the literature [26] with some modifications. After 28 days of administration, the mice were anaesthetized using 10% chlorine hydrate. Anaesthetized mice were fixed on a clean foam board, and the skin around the neck was sterilized using alcohol-soaked cotton swabs. The skin of the mouse neck was cut, the neck fat was separated, and the mouse bronchi were located. An indwelling needle was inserted into the mouse bronchus and fixed using cotton thread. Then, 1 mL of normal saline was drawn continuously and slowly 3 times through the indwelling needle to the lung, and the final normal saline was collected as BALF. The recovery rate was greater than 80%.

### H&E and Masson’s trichrome staining

The collected left lower lung from mice was fixed in 4% paraformaldehyde for one week, dehydrated and embedded in paraffin. The embedded tissue was cut into 3–5 μm thick sections. The tissue sections were stained separately according to the instructions of H&E staining and Masson trichrome staining (BASO, BA-40798). A Panoramic MIDI II 3DHISTECH digital pathology system was used to acquire section images. CaseViewer software was used for processing. Fibrosis was scored as previously described[27]. Fibre volume fractions were determined using ImageJ.

### Hydroxyproline assay

A hydroxyproline determination kit (#A030-2-1, Nanjing Jiancheng Institute of Biological Engineering, Nanjing, China) was used to analyse the hydroxyproline content of lung tissue samples. In brief, tissue was hydrolysed and neutralized using sodium hydroxide. Then, chloramine-T and dimethylaminobenzaldehyde were sequentially added. Finally, a spectrophotometer was used to determine the absorbance at 550 nm to evaluate hydroxyproline content in the lung tissue. Results are expressed as μg/mg wet lung tissue.

### Immunohistochemical (IHC) staining

IHC staining was performed on lung tissue sections using an IHC staining kit (ZSGB-BIO Co., Beijing, China) as previously described [28]. Paraffin-embedded lung sections were stained with primary antibodies (α-SMA, Collagen I and p-Stat3 (TY705)) using a DAB Detection Kit (ZSGB-BIO Co., Beijing, China).

### Quantification of immune cells in the lung tissue

Lung tissue was disrupted according to a previously described method and lysed with collagenase (37 °C for 90 min) [28]. Changes in the cell ratio were assessed by flow cytometry (BD LSR II) after incubation with different antibodies. The data were analysed using FlowJo software.

#### ELISA

Levels of TNF-α, IL-6 and other factors in the BALF were analysed by ELISA using commercially available kits (RAB0477, RAB0308, Merck Millipore, Billerica, MA, USA) according to the manufacturer’s instructions. Levels of TNF-α, IL-2 and other factors in murine serum were analysed by ELISA using commercially available kits (#560,484, Cell Signaling Technology Company, MA, USA) according to the manufacturer’s instructions.

### Statistical analysis

The data are presented as the mean ± SD of three independent experiments. Unpaired two-tailed Student’s t tests were used to compare two groups, and one-way ANOVA was performed for multiple group comparisons followed by Tukey’s test. *P*-values < 0.05 were considered statistically significant; individual *P*-values are indicated by asterisks in the figures: *P < 0.05, **P < 0.01, ***P < 0.001; ^#^P < 0.05, ^##^P < 0.01 and ^###^P < 0.001. Statistical analysis was performed using GraphPad Prism 6.0 (GraphPad software, San Diego, CA, USA).

## Results

### NIF combines with Stat3

**Fig. 1:**
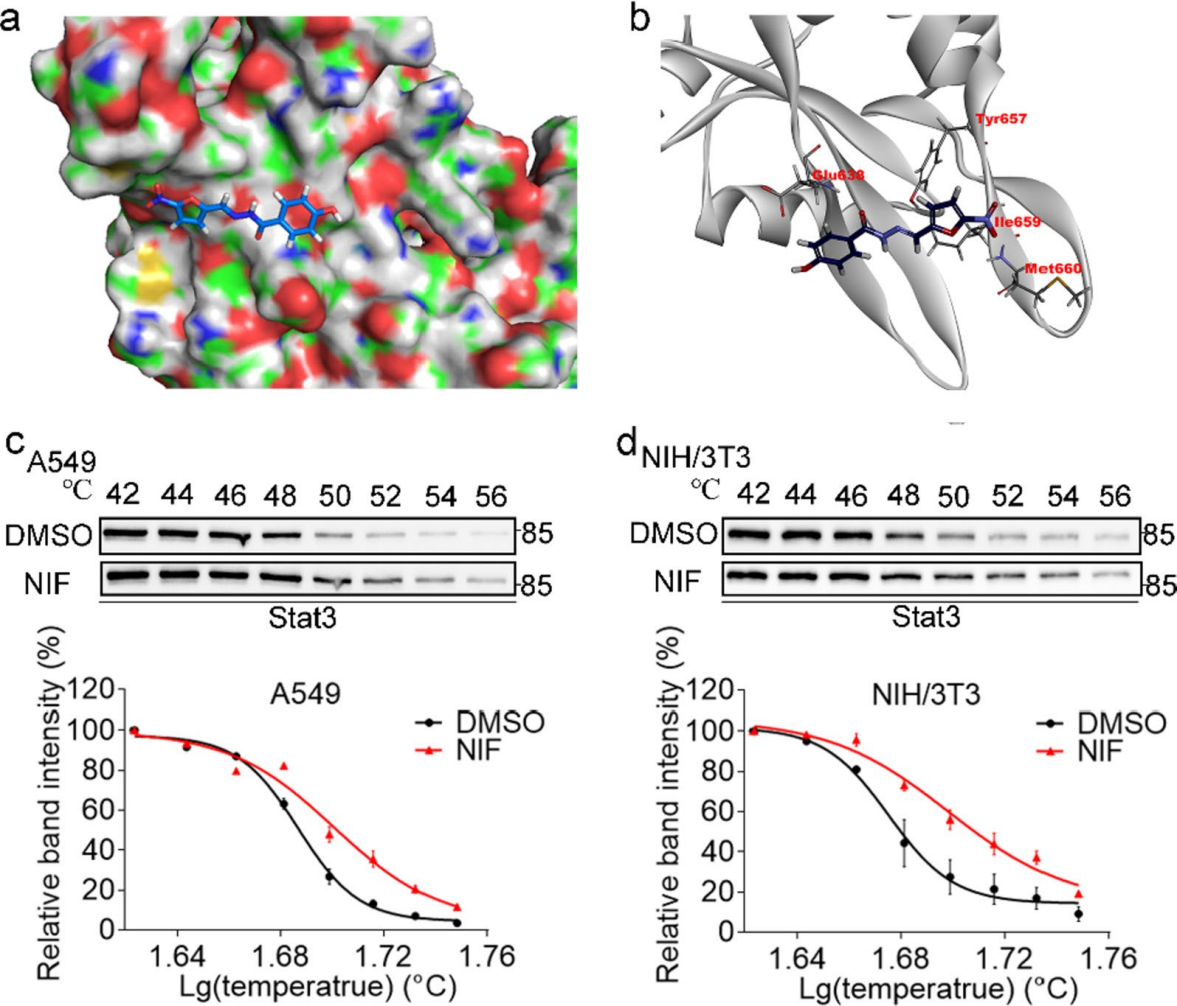
NIF combines with Stat3. **a** Surface of the electrostatic map. **b** Residues of Stat3. **c**, **d** Upper panel: representative western blots showing the effects of NIF (200 μM) on thermal stabilization of Stat3 protein in A549 and NIH/3T3 cells. Lower panel: Stat3 degradation curve with temperature. Results are expressed as the mean ± SD of three independent experiments per condition

Although NIF has previously been reported to be a potential inhibitor of Stat3 [15], we verified this using molecular docking. As shown in Fig. 1a, b, NIF forms intermolecular hydrogen bonds with GLU638 and MET660 of the SH2 domain, stabilizing the complex. Moreover, the benzene ring moiety forms a conjugation with TYR657 and ILE659. Together, these interactions promote the binding of NIF to Stat3. The docking results revealed that the interaction energy score between NIF and Stat3 was 22.4538, indicating that NIF stably binds to Stat3. In addition, as shown in Fig. 1c, d, Stat3 is gradually degraded with increasing temperature, but compared to DMSO, NIF slowed its degradation rate in A549 and NIH/3T3 cell lines, with the temperature required for half Stat3 degradation in the NIF group increasing by 1.8 ℃ and 2.61 ℃ in A549 and NIH/3T3 cells, respectively. It was further confirmed that NIF could binds to Stat3 in these cell lines, indicating that it is a potential inhibitor of Stat3.

### NIF reduces the inflammatory response in BLM-induced pulmonary fibrosis in vivo

**Fig. 2:**
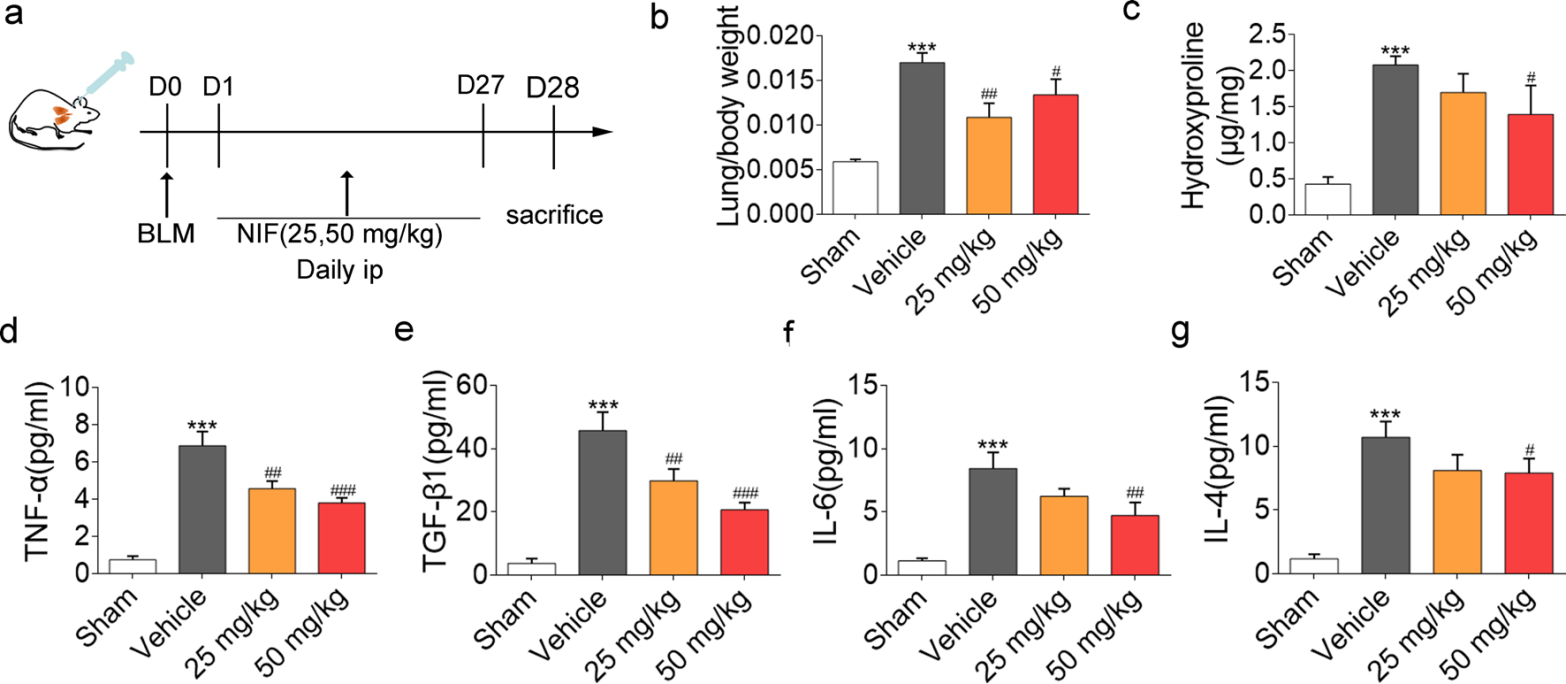
NIF reduces inflammation and expression of fibrotic markers in BLM-induced lung fibrosis. **a** Experimental outline for NIF preventing fibrosis after BLM-induced lung injury in mice: n = 10 (Sham); n = 14 (Vehicle); n = 10 (NIF 25 mg/kg); n = 14 (NIF 50 mg/kg). **b** Lung weight coefficient of mice depicted in **a**. **c** Content of hydroxyproline in lung homogenates from the groups of mice depicted in **a**. **d**–**g** Analysis of TNF-α, IL-4, IL-6, and TGF-β1 levels in BALF from control and BLM-treated mice in the presence of vehicle and NIF. Statistical significance was evaluated using one-way ANOVA followed by Tukey’s test. All data are shown as the mean ± SD; n = 3–5 per group; ***P < 0.001 vs. Sham; ^#^P < 0.05, ^##^P < 0.01, ^###^P < 0.001 vs. Vehicle

First, we evaluated the effect of NIF on BLM-induced pulmonary fibrosis in mice. According to our previous research on NIF [14, 28], intraperitoneal administration (25 mg/kg or 50 mg/kg) was selected for the in vivo experiments. As shown in Fig. 2a, we administered NIF to mice beginning on the first day of bronchial instillation of BLM (~ 2.5 mg/kg) for a total of 27 days. After 28 days, BLM induced severe pulmonary fibrosis in mice, resulting in a significant increase in the lung weight coefficient. Compared to the Vehicle, NIF reduced the lung weight coefficient (Fig. 2b).

Furthermore, NIF attenuated the BLM-induced increase in hydroxyproline levels (Fig. 2c). Simultaneously, expression of the proinflammatory cytokines IL-6, IL-4, TNF-α and TGF-β1 in BALF samples was decreased in response to NIF treatment (Fig. 2d–g).

### NIF inhibits pulmonary fibrosis induced by BLM

**Fig. 3:**
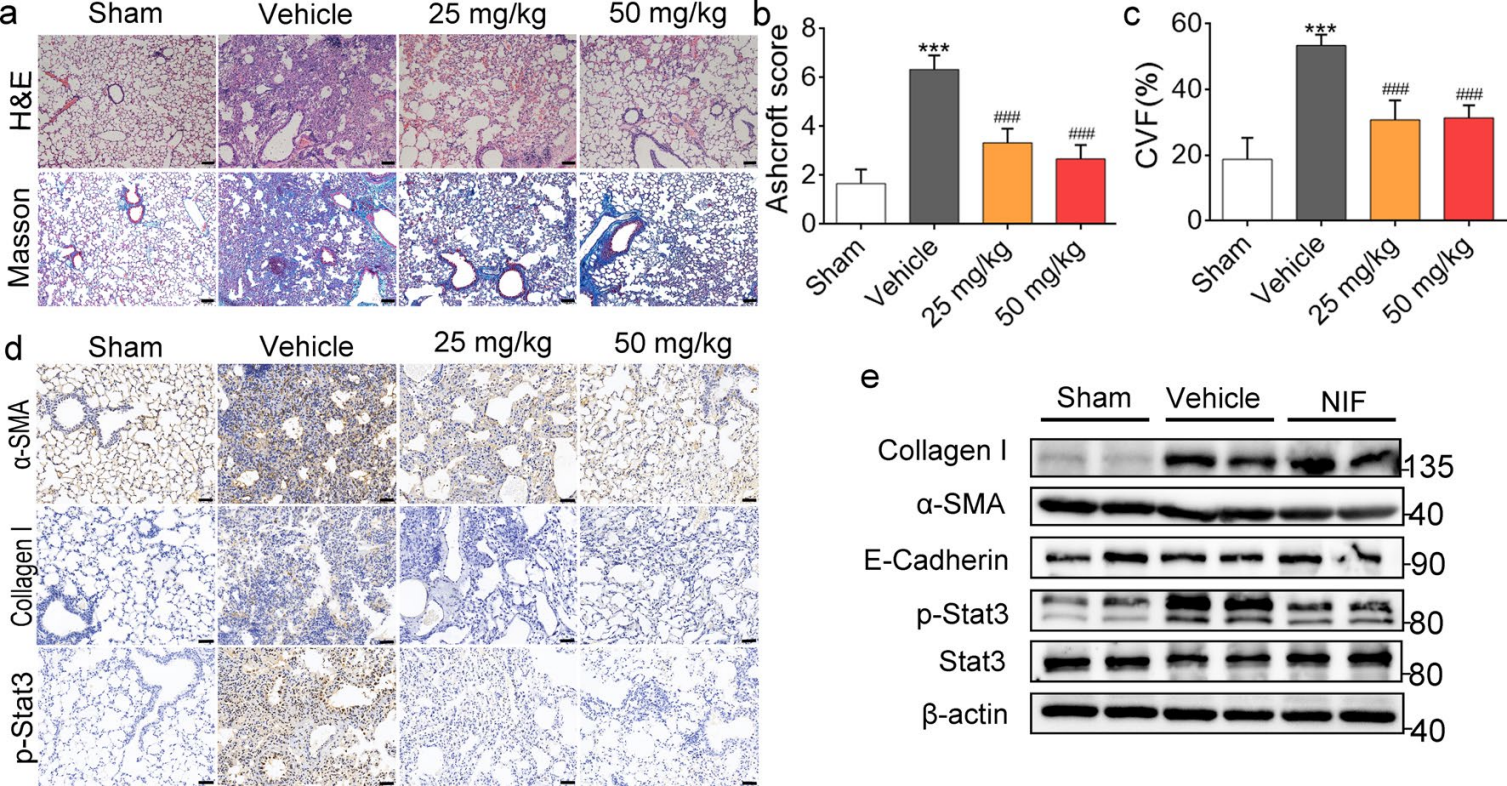
NIF inhibits pulmonary fibrosis induced by BLM. **a** Representative images showing H&E and Masson’s trichrome staining. Scale bars, 100 μm. **b** Quantification of fibrosis on lung sections based on the results of H&E staining; n = 5. **c** Quantification of collagen content volume fraction of lung sections based on Masson staining results; n = 5. **d** Representative images showing α-SMA, Collagen I and p-Stat3 staining of lung sections in mice. Scale bars, 50 µm. **e** Representative immunoblots of Collagen I, α-SMA, E-Cadherin, p-Stat3, Stat3 and β-actin in lung homogenates from mice as indicated. NIF (50 mg/kg). Statistical significance was evaluated using one-way ANOVA followed by Tukey’s test; All data are shown as the mean ± SD, ***P < 0.001 vs. Sham; ^###^P < 0.001 vs. Vehicle

As shown by H&E and Masson staining in Fig. 3a, NIF alleviated BLM-induced structural damage and collagen proliferation in the lung and decreased the fibrosis score (Fig. 3b) and collagen volume fraction (CVF%) (Fig. 3c), indicating that NIF reduced collagen proliferation and improved pulmonary interstitial fibrosis. α-SMA and Collagen I are important proteins for characterizing fibrosis. As shown in Fig. 3d, e, and Additional file 1: Fig. S2, NIF inhibited the expression of α-SMA and Collagen I induced by BLM. Moreover, NIF inhibited EMT in vivo. As shown in Fig. 3e and Additional file 1: Fig. S2, NIF inhibited the decrease in E-Cadherin (epithelial marker) expression. This suggests that NIF inhibits BLM-induced pulmonary fibrosis and EMT in vivo. At the same time, we found that BLM induced abnormal activation of Stat3 (Fig. 3d, e; Additional file 1: Fig. S2), while this abnormal activation was decreased in response to NIF, indicating that NIF may inhibit pulmonary fibrosis by blocking abnormal Stat3 activation.

### NIF regulates the immune microenvironment in the lung

**Fig. 4:**
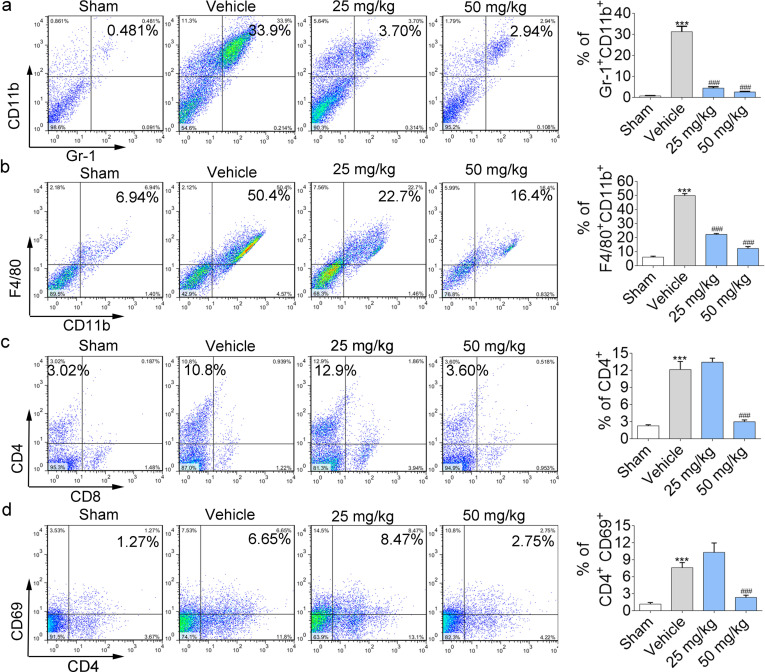
NIF regulates the immune microenvironment of the lungs. **a**–**d** Quantification of MDSCs, macrophages, and CD4^+^, CD4^ +^ CD69^ +^ T lymphocytes infiltration in the lung tissue. Statistical significance was assessed using one-way ANOVA followed by Tukey’s test. All data are shown as the mean ± SD. n = 3; ***P < 0.001 vs. Sham; ^###^P < 0.001 vs. Vehicle

During the early stage of fibrosis, the innate immune system in the body is primarily antagonistic to stimulation, while immune cells in damaged tissues also contribute to chronic inflammation and tissue remodelling by secreting growth factors, cytokines, and chemokines and activating extracellular matrix synthesis [29, 30]. Therefore, strategies against immunosuppressive cells and cytokines may be very beneficial for tissue fibrosis during chronic inflammation. We found that BLM induced an increase in the expression of immune cells, inducing Gr-1^+^ CD11b^+^ (MDSCs), F4/80^+^ CD11b^+^ (macrophages), and CD4^+^, CD4 ^+^ CD69^ +^ T lymphocytes. NIF reduced the number of these immune cells to closer to normal levels (Fig. 4a–d).

### NIF reverses pulmonary fibrosis induced by BLM

**Fig. 5:**
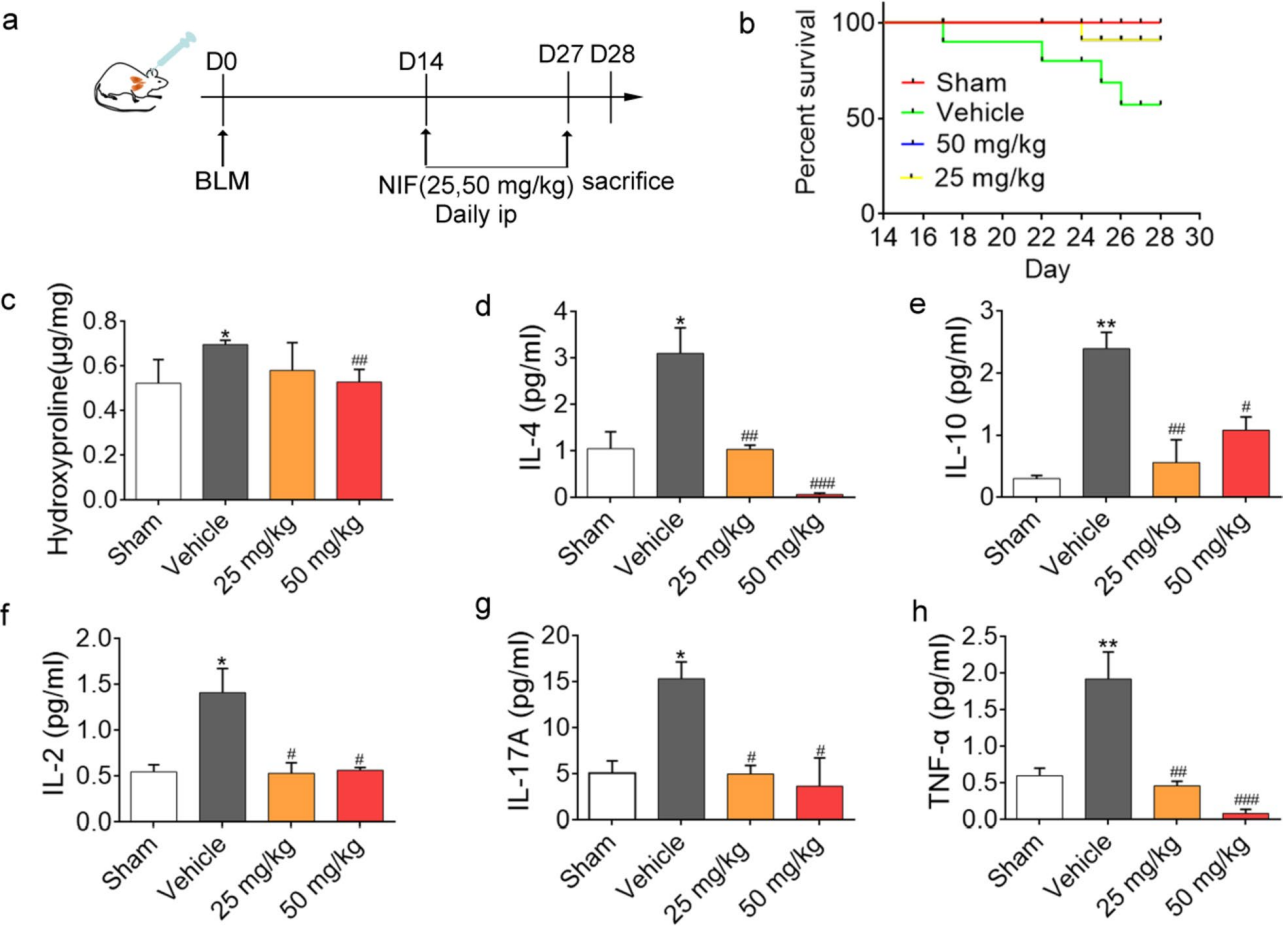
NIF promotes the resolution of inflammation and improves survival in BLM-induced lung fibrosis. **a** Experimental design of NIF for the treatment of fibrosis after BLM-induced lung injury in mice. **b** Survival curves of mice: n = 4 (Sham), n = 9 (Vehicle), n = 8 (25 mg/kg, 50 mg/kg). **c** Hydroxyproline content in lung homogenates from the groups of mice depicted in **a**. Statistical significance was assessed using Student’s t test. **d**–**h** Analysis of IL-2, IL-4, IL-10, IL-17A, and TNF-α levels in the serum of control and BLM-treated mice in the presence of vehicle or NIF. Statistical significance was assessed using one-way ANOVA followed by Tukey’s test. All data are shown as the mean ± SD; n = 3–5; *P < 0.05, **P < 0.01 vs. Sham; ^#^P < 0.05, ^##^P < 0.01, ^###^P < 0.001 vs. Vehicle

**Fig. 6:**
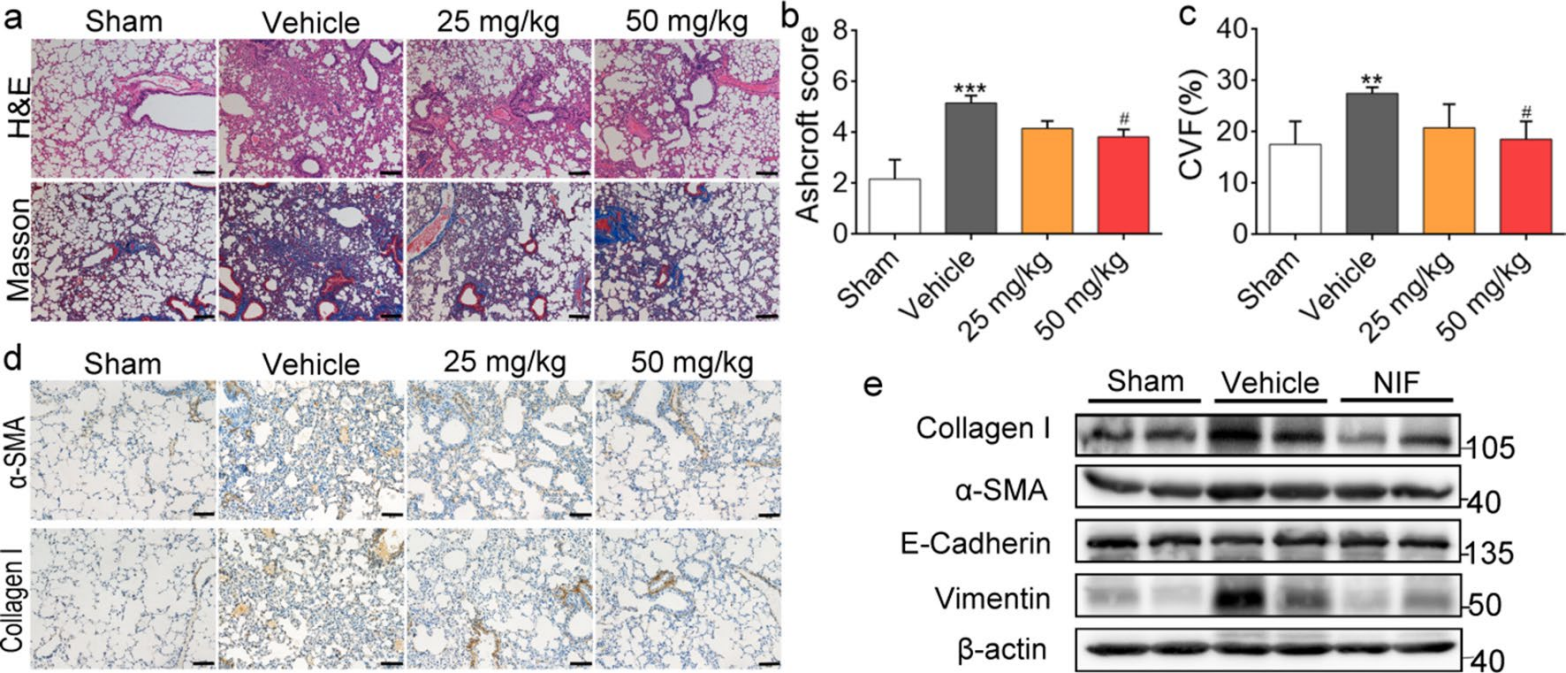
NIF reverses pulmonary fibrosis induced by BLM. **a** Representative images showing H&E and Masson’s trichrome staining. Scale bars, 100 μm. **b** Quantification of fibrosis in lung sections based on the results of H&E staining. **c** Quantification of the collagen content volume fraction of lung sections based on Masson staining results. **d** Representative images showing α-SMA and Collagen I staining of lung sections in mice. Scale bars, 50 µm. **e** Representative immunoblots of α-SMA, Collagen I, E-Cadherin, Vimentin, and β-actin in lung homogenates of mice as indicated. NIF (50 mg/kg). Statistical significance was assessed using one-way ANOVA followed by Tukey’s test. All data are shown as the mean ± SD; n = 3–5; **P < 0.01, ***P < 0.001 vs. Sham; ^#^P < 0.05 vs. Vehicle

To further determine the therapeutic effect of NIF on pulmonary fibrosis, we also examined, 

the effects of delayed NIF treatment (25 mg/kg or 50 mg/kg), that is, NIF treatment beginning 14 days after injury induced by BLM (~ 1 mg/kg) for a total of 14 days (Fig. 5a). The results showed that NIF prolonged the survival rate of mice (Fig. 5b) and reduced the content of hydroxyproline (Fig. 5c). Of note, the levels of IL-2, IL-4 and other factors in the mouse serum were further reduced by NIF (Fig. 5d–h). Importantly, as shown in Fig. 6a, NIF stimulation reversed BLM-induced lung inflammation and collagen expression. Changes in the fibrosis score and CVF% were further confirmed (Fig. 6b, c). Additionally, the results of immunohistochemistry and protein analysis demonstrated that NIF reduced the expression of α-SMA and Collagen I and inhibited EMT (Fig. 6d, e; Additional file 1: Fig. S3). These results suggest that NIF promotes fibrosis resolution even when treatment is delayed.

### NIF inhibits the activation of fibroblasts

**Fig. 7:**
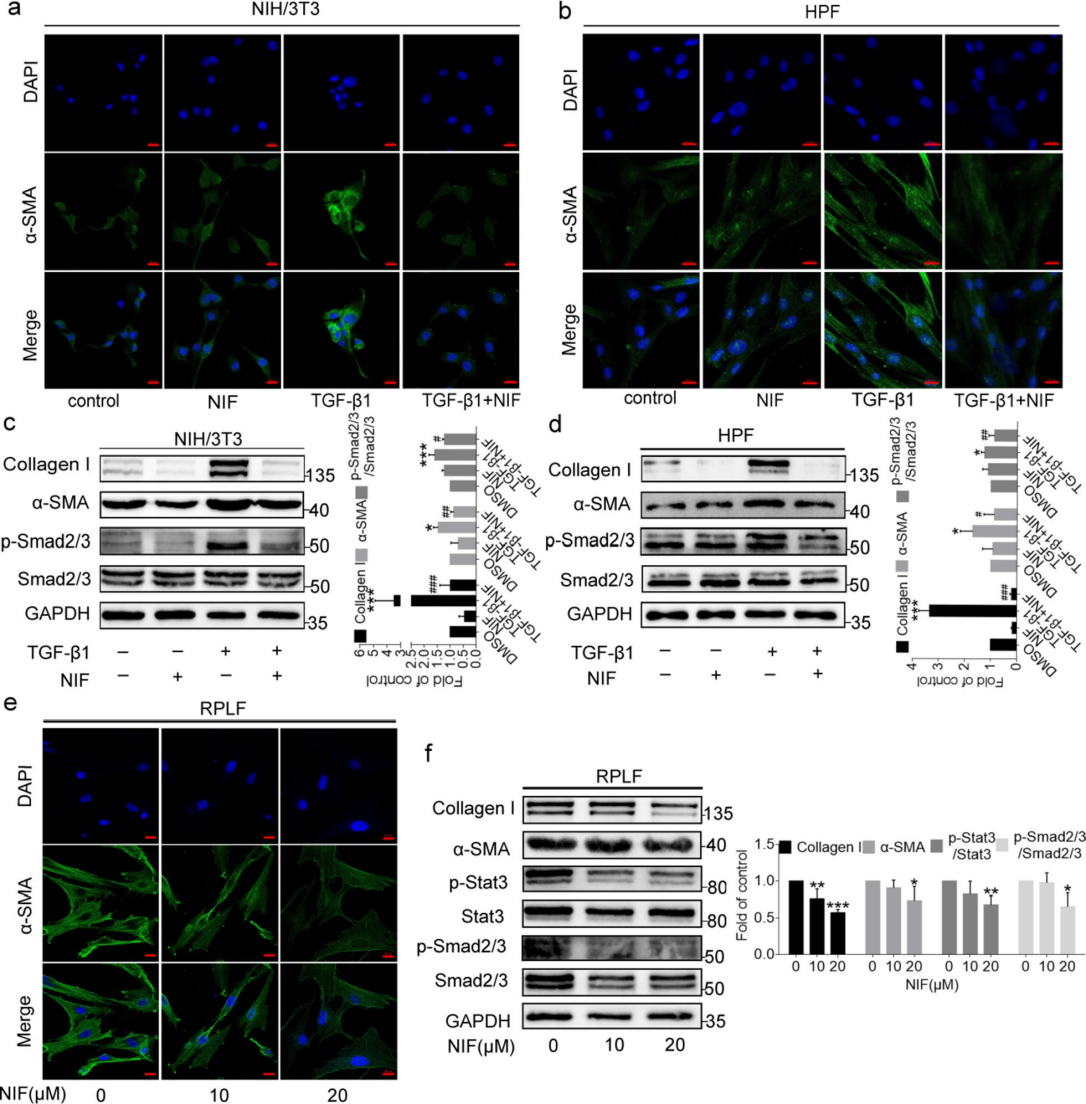
NIF inhibits fibroblast activation. **a**, **b** Representative fluorescence staining images of α-SMA (green) and nuclei (blue) in NIH/3T3 and HPF cells treated with TGF-β1 or NIF for 24 h. Scale bars, 20 µm. **c**, **d** Left panel: representative western blot of α-SMA, Collagen I, p-Smad2/3, Smad2/3, and GAPDH from NIH/3T3 and HPF cells treated with TGF-β1 or NIF for 24 h. Right panel: bar graph showing the mean data from all subjects analysed in each group. **e** Representative fluorescence staining images of α-SMA (green) and nuclei (blue) in RPLFs stimulated with NIF (24 h). Scale bars, 20 µm. **f** Left panel: representative western blot of Collagen I, α-SMA, p-Stat3, Stat3, p-Smad2/3, Smad2/3, and GAPDH in RPLFs stimulated with NIF (24 h). Right panel: bar graph showing the mean data from all subjects analysed in each group. Statistical significance was assessed using one-way ANOVA followed by Tukey’s test. All data are shown as the mean ± SD of three independent experiments per condition; *P < 0.05; **P < 0.01; ***P < 0.001 vs. DMSO; ^#^P < 0.05, ^##^P < 0.01, ^##^P < 0.001 vs. TGF-β1

Based on these findings, we further explored the potential mechanism of NIF and examined the effect of NIF on the activation of fibroblasts induced by TGF-β1. First, the MTT results showed that NIF inhibited the proliferation of pulmonary fibroblasts in a concentration-dependent manner (Additional file 1: Fig. S4). Next, we starved fibroblasts for 6 h, stimulated them with TGF-β1, and analysed the increase in the fibrosis phenotype at the protein level using immunofluorescence and western blot techniques. As shown in Fig. 7a–d, α-SMA and Collagen I were enhanced by stimulation with TGF-β1, consistent with previous reports[31]. Upon treatment with NIF, the increases in α-SMA and Collagen I induced by TGF-β1 were reduced (Fig. 7a–d), indicating that NIF significantly suppresses the activation of fibroblasts induced by TGF-β1. In addition, NIF decreased TGF-β1 stimulation-induced expression of p-Smad2/3 (Fig. 7c, d), further demonstrating that NIF inhibits TGF-β pathways.

To further examine the antifibrotic effects of NIF on activated lung fibroblasts, we isolated lung fibroblasts from rats treated with BLM[32]. The results showed that NIF inhibited the proliferation of activated lung fibroblasts in a concentration-dependent manner (Additional file 1: Fig. S4). Expression of α-SMA and Collagen I was also decreased in a concentration-dependent manner in response to NIF treatment (Fig. 7e, f). Furthermore, in primary activated pulmonary fibroblasts, p-Stat3 is elevated [33], and NIF decreased expression of p-Stat3 and p-Smad2/3 in a concentration-dependent manner (Fig. 7f). These results indicate that NIF may inhibit the TGF-β/Smad and Stat3 pathways and the activation of fibroblasts.

### NIF suppresses EMT and migration of A549 cells

**Fig. 8:**
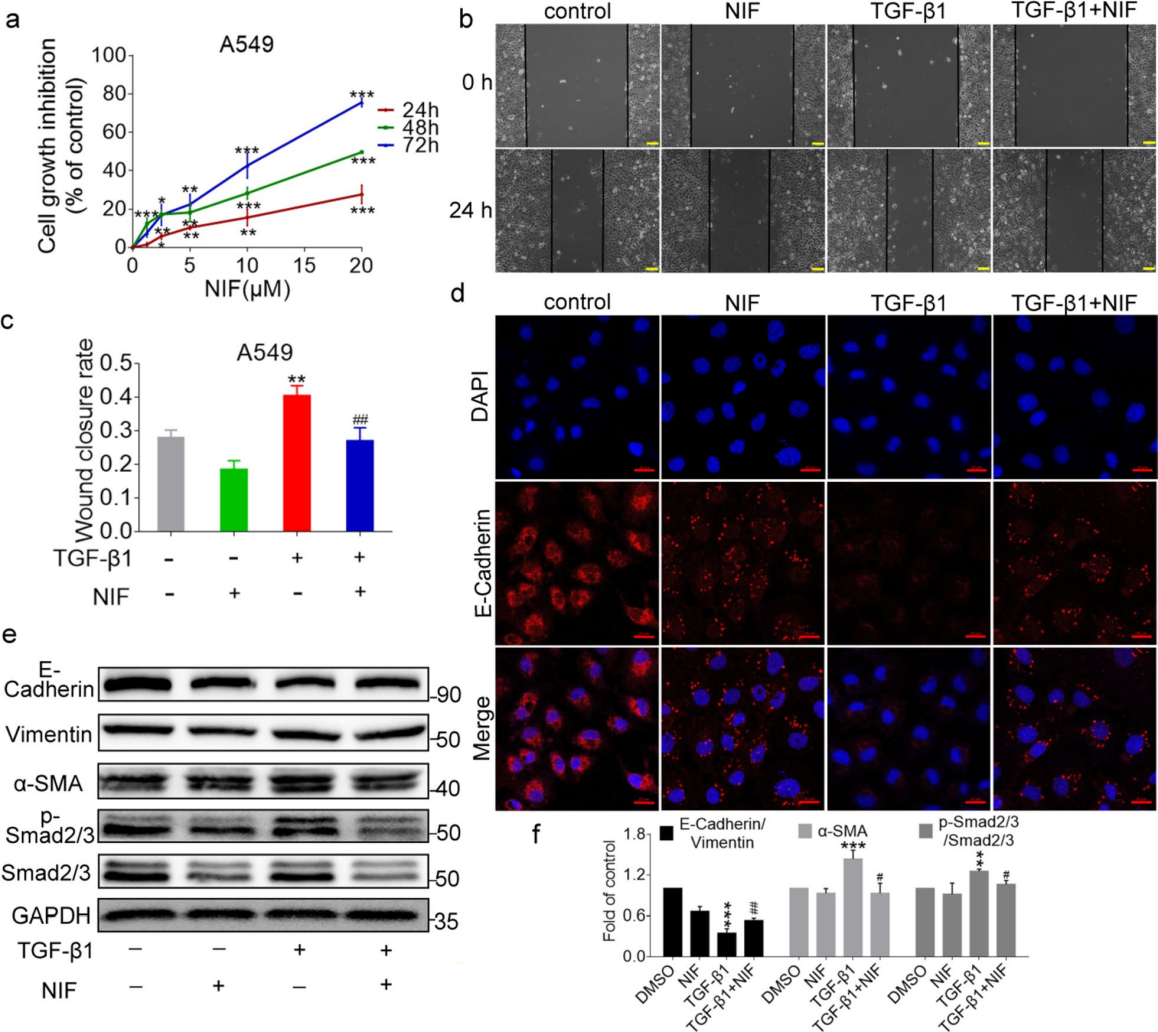
NIF suppresses EMT and migration of A549 cells induced by TGF-β1. **a** Viability of A549 cells after treatment with different concentrations of NIF for 24, 48 or 72 h. Statistical significance was assessed using Student’s t test; each point represents the mean ± SD of three independent experiments per condition; *P < 0.05; **P < 0.01; ***P < 0.001 vs. vehicle control. **b** Representative images of migration in A549 treated with TGF-β1 or NIF for 24 h. Scale bars, 100 µm. **c** Quantified wound healing area ratio. **d** Representative fluorescence staining images of E-Cadherin (red) and nuclei (blue) in A549 cells treated with TGF-β1 or NIF for 24 h. Scale bars, 20 µm. **e** Representative immunoblots of α-SMA, E-Cadherin, Vimentin, p-Smad2/3, Smad2/3, and GAPDH from A549 cells treated with TGF-β1 or NIF for 24 h. **f** Bar graph showing the mean data from all subjects analysed in each group in **e**. Statistical significance was assessed using one-way ANOVA followed by Tukey’s test in **c**, **f**. All data are shown as the mean ± SD of three independent experiments per condition; **P < 0.01; ***P < 0.001 vs. DMSO; ^#^P < 0.05, ^##^P < 0.01 vs. TGF-β1

When local fibroblasts are inadequate for tissue repair and remodelling, pulmonary epithelial cells that undergo EMT are a source of myofibroblast cells that can promote the occurrence and development of pulmonary fibrosis, and TGF-β1 can induce EMT and migration of lung epithelial cells in vitro [34–36]. Therefore, we used TGF-β1 to stimulate A549 cells, which caused them to undergo migration and EMT. As shown by MTT, NIF inhibited the proliferation of A549 cells in a time- and concentration-dependent manner (Fig. 8a). As shown in Fig. 8b, c, TGF-β1 induced the migration of A549 cells compared to the control group, but the induction of migration by TGF-β1 was inhibited after NIF administration. These results demonstrate that NIF inhibits the migration of epithelial cells.

With respect to EMT, Fig. 8d–f shows that TGF-β1 stimulation decreased the expression of E-Cadherin (epithelial marker) in A549 cells and increased Vimentin (interstitial marker) and α-SMA expression. NIF inhibited the TGF-β1-induced decrease in E-Cadherin and the increase in Vimentin and α-SMA. These results show that NIF reduces TGF-β1-induced EMT in A549 cells. We also assessed the activation of Smad2/3 in A549 cells and fibroblasts and found that NIF inhibited TGF-β1-induced expression of p-Smad2/3 (Fig. 8e, f). These results further suggest that the reduction in pulmonary fibrosis induced by NIF in vitro may be related to inhibition of abnormally activated Smad2/3.

## Discussion

The potential mechanisms underlying specific types of pulmonary fibrosis are difficult to unravel. However, myofibroblasts serve as the primary effector of pulmonary fibrosis, and their role in the development of pulmonary fibrosis has been increasingly recognized. At present, the most important sources of myofibroblasts are thought to be the transformation of resident fibroblasts, the differentiation of circulating bone marrow-derived progenitor cells and the EMT of epithelial-derived cells [37]. Thus, we mimicked the activation of fibroblasts and EMT of epithelial cells by administering TGF-β1 in vitro. When activated, fibroblasts exhibited phenotypes such as those characterized by the expression of α-SMA and Collagen I (Fig. 7) [38]. In a model of BLM-induced pulmonary fibrosis in mice, expression levels of α-SMA and Collagen I were also significantly increased (Figs. 3 and 6). Herein, NIF was shown to inhibit the production of α-SMA and Collagen I induced by TGF-β1 and BLM both in vitro and in vivo. The reduction in α-SMA and Collagen I in BLM-induced primary myofibroblasts also confirmed the inhibitory effect on myofibroblasts (Fig. 7e, f). These results suggest that NIF suppresses the origin of myofibroblasts and the deposition of ECM components by inhibiting the transformation of fibroblasts into myofibroblasts, achieving partial inhibition of pulmonary fibrosis.

In pulmonary fibrosis, EMT is a process in which pulmonary epithelial cells undergo phenotypic transformation into mesenchymal cells, typically fibroblasts and myofibroblasts, and participate in the formation of pulmonary fibrosis [39, 40]. Our data demonstrated that TGF-β1 stimulates EMT in A549 cells, which adopted the characteristics of myofibroblasts, that is, spindle-shaped morphology and expression of various mesenchymal immune cytochemicals, such as α-SMA and Collagen I (Fig. 8). These substances are key mediators of ECM, structural remodelling, and the destruction of alveolar capillary units during and after lung injury [41]. NIF inhibited these changes after A549 cells were stimulated with TGF-β1, thereby reducing the number of epithelial-derived myofibroblasts and expression of some types of collagen and ECM proteins (Fig. 8). These results were also confirmed in vivo (Figs. 3 and 6). More importantly, there are data showing that the alveolar epithelium is one of the sources of TGF-β1 during lung injury and fibrosis and that TGF-β1 in turn regulates the function and differentiation of fibroblasts, which further aggravates the development of pulmonary fibrosis [42]. Therefore, inhibiting EMT in epithelial cells largely inhibits pulmonary fibrosis.

In pulmonary fibrosis, in addition to myofibroblasts, certain immune cells are also involved in the development of disease, and congenital and adaptive immunity contribute to fibrogenesis in many organs [43]. Among these immune cells, macrophages are key regulators of fibrosis that are usually found in the vicinity of collagen-producing myofibroblasts and can secrete multiple profibrotic soluble mediators, chemokines and matrix metalloproteinases, such as TGF-β1 [44–46]. The depletion of macrophages may attenuate pulmonary fibrosis. MDSCs are highly expressed in IPF patients and are inversely correlated with lung function in IPF, indicating that controlling MDSC expansion and accumulation represents a promising treatment for IPF [47]. T lymphocyte accumulation is most pronounced in the lung tissue, which undergoes intertissue fibrosis and honeycomb changes, while there are only a few T cells in the area of relatively normal tissue [48]. CD4^+^ T lymphocytes specialize in the production of soluble factors (cytokines), which may play a role in fibrosis (IL-4, IL-13) [49]. In addition, inflammatory factors also regulate the TGF-β1-mediated pathway, and the proinflammatory cytokine TNF-α enhances TGF-β1-induced EMT by upregulating the TGF-β receptor type I [50]. The results of this study found that NIF attenuates the BLM-induced imbalance in macrophages, MDSCs and T lymphocytes in lung tissue. In addition, NIF also inhibited the expression of inflammatory factors in BALF and serum. These results demonstrate that NIF also attenuates BLM-induced pulmonary fibrosis through immunosuppressive effects.

In a variety of signalling pathways involving pulmonary fibrosis, activation of the TGF-β signalling pathway is considered very important. The downstream signal of TGF-β1 is significantly activated in both BLM-injured mice and TGF-β1-stimulated cells, as indicated by increased phosphorylation of Smad2/3, which is an essential mechanism in the pathogenesis of pulmonary fibrosis [51]. In addition, activation of Smad2/3 promotes fibroblast proliferation and differentiation [52]. The role of NIF in this mechanism was supported by the data herein, which demonstrated that NIF reduced Smad2/3 phosphorylation in fibroblasts and epithelial cells. In addition, macrophages are the primary source of TGF-β1 [53], and flow cytometry revealed that NIF regulates the content of macrophages in lung tissue, further confirming that NIF inhibits the TGF-β/Smad pathway. In addition to the TGF-β/Smad pathway, JAK/Stat is one of the most important pathways in the TGF-β/non-Smad pathway [54]. A large amount of evidence has shown that Stat3 plays an important role in the occurrence and development of pulmonary fibrosis [33, 55]. Inhibition of Stat3 weakens the sensitivity of IPF patients to exogenous TGF-β1, further blocking the transformation of fibroblasts into myofibroblasts induced by TGF-β1 and the release of collagen from fibroblasts, improving two types of skin fibrosis mouse models [56, 57]. In this study, we verified the combination of NIF and Stat3 and found that NIF inhibited Stat3 activation in vitro and in vivo.

## Conclusion

Overall, in this study, we demonstrated that NIF ameliorates and reverses pulmonary fibrosis, but a more detailed understanding of this mechanism is worth investigating. There are still some shortcomings in the in vivo administration and dosage of NIF that need to be improved. There is currently no good treatment for pulmonary fibrosis, and our findings have revealed some strategies for its treatment.

## Supplementary Information

**Additional file 1.** Additional figures.

## Data Availability

We would like to share part of our data, because some of our data will be used in future research.

## Abbreviations

IPF: Idiopathic pulmonary fibrosis
BLM: Bleomycin
NIF: Nifuroxazide
TGF-β1: Transforming growth factor-β1
EMT: Epithelial-mesenchymal transition
Stat3: Signal transduction and activator of transcription 3
HPF: Human pulmonary fibroblasts
RPLF: Rat primary lung fibroblasts
IL-6: Interleukin-6
IL-4: Interleukin-4
IL-2: Interleukin-1β2
IL-17A: Interleukin-17A
IL-10: Interleukin-10
TNF-α: Tumor necrosis factor α
ELISA: Enzyme-linked immunosorbent assay
MDSCs: Myeloid-derived suppressor cells
DAPI: 4′,6-Diamidino-2-phenylindole
FBS: Fetal bovine serum
IHC: Immunohistochemistry
PBS: Phosphate-buffered saline
BALF: Bronchoalveolar lavage fluid
FVC: Forced vital capacity
α-SMA: Alpha-smooth muscle actin
CVF: Collagen volume fraction
ECM: Extracellular matrix
