# Supplementary material for: Nifuroxazide ameliorates pulmonary fibrosis by blocking myofibroblast genesis: a drug repurposing study
Source: Respir Res. 2022 Feb 16;23:32. doi: 10.1186/s12931-022-01946-6 (PMC8848910; doi:10.1186/s12931-022-01946-6)
Supplement: Supplementary file 1 — Additional file 1. Additional figures. [file 12931_2022_1946_MOESM1_ESM.docx]

**Nifuroxazide ameliorates pulmonary fibrosis by blocking myofibroblast genesis: A drug repurposing study**

Cailing Gan^1,#^, Qianyu Zhang^2,#^, Hongyao Liu^1^, Guan Wang^1,3,4^, Liqun Wang^2^, Yali Li^2^, Zui Tan^1^, Wenya Yin^2^, YuqinYao^2^, Yongmei Xie^1^, Liang Ouyang^1^, Luoting Yu^1^, Tinghong Ye^1,*^

^1^ Sichuan University-Oxford University Huaxi Gastrointestinal Cancer Centre, State Key Laboratory of Biotherapy , West China Hospital, Sichuan University, Chengdu 610041, China;

^2^ Department of Nutrition and Food Hygiene, School of Public Health, West China Medical School, Sichuan University, Chengdu 610041, China;

^3^ Innovation Center of Nursing Research, West China Hospital, Sichuan University, Chengdu 610041, China;

^4^ Nursing Key Laboratory of Sichuan Province, Sichuan University, Chengdu 610041, China

^#^ Cailing Gan and Qianyu Zhang contributed equally to this work.

*Corresponding author: ^1^ Sichuan University-Oxford University Huaxi Gastrointestinal Cancer Centre, State Key Laboratory of Biotherapy , West China Hospital, Sichuan University, Chengdu 610041, China, 17# 3rd Section, Ren Min South Road, 610041 Chengdu, China. Tel.:+862885503817; fax: +86 2885164060. [yeth1309@scu.edu.cn](mailto:yeth1309@scu.edu.cn). (Tinghong Ye).

The authors confirm that they have no conflict of interest.


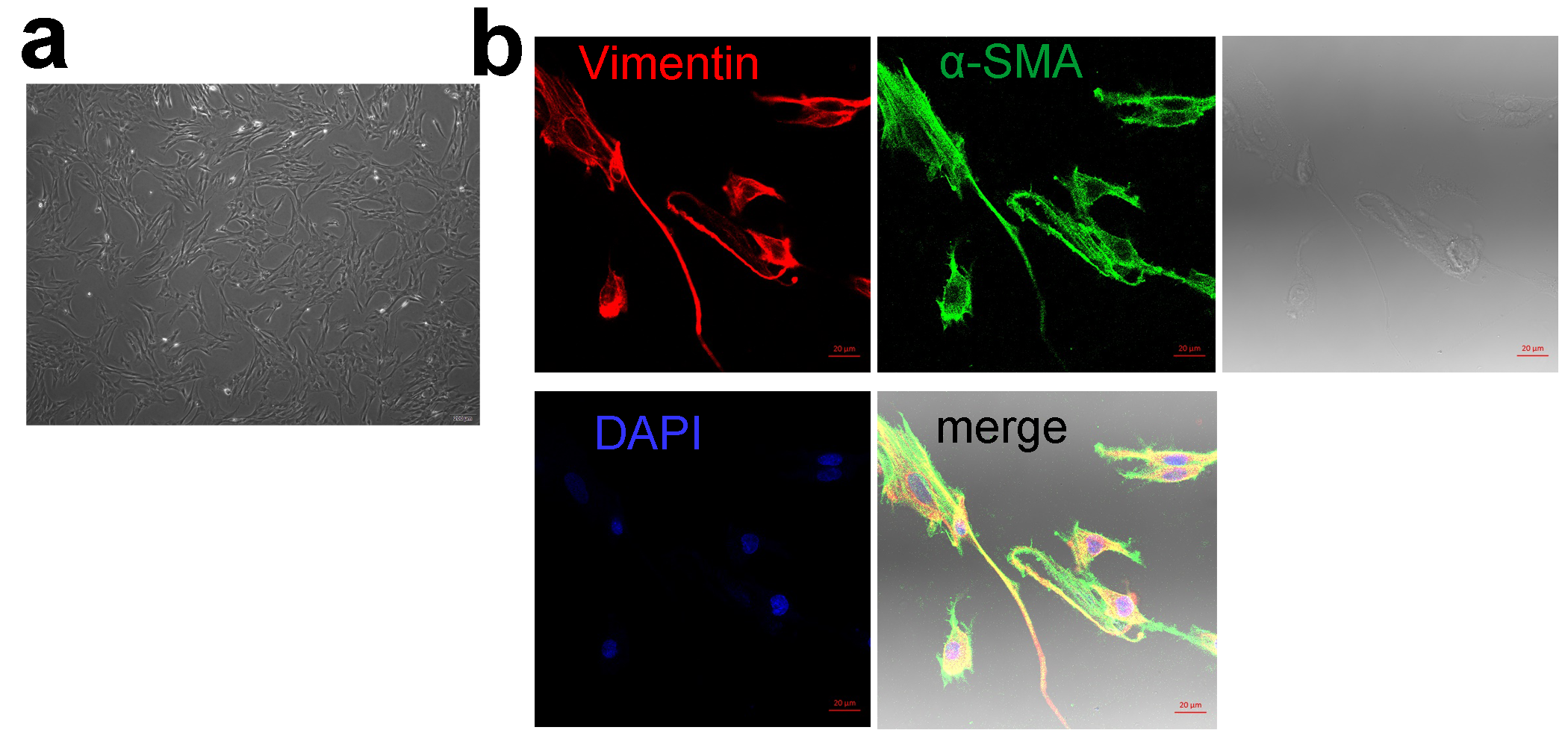
Figure S1 Characterization of primary human lung fibroblasts. (a) All primary human lung fibroblasts cells displayed typical spindle-shaped morphology under a light microscopy (Scar bar, 100 μm). (b) Indirect immunofluorescence staining of primary human pulmonary fibroblasts for vimentin and α-smooth muscle actin (α-SMA). Scar bars, 20 μM.


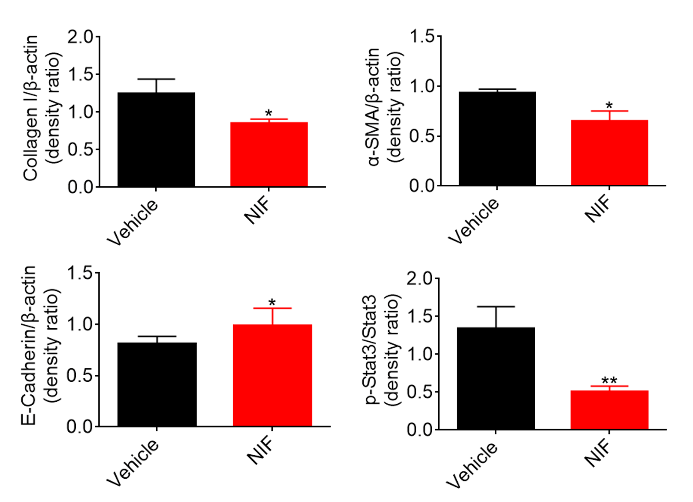


Figure S2 Bar graph showing the mean data from all subjects analyzed in each group in Fig 3e. Statistical significance was tested using Student’s t test; each point represents the mean ± SD for at least 3 independent experiments (*P < 0.05; **P < 0.01 vs. Vehicle control).


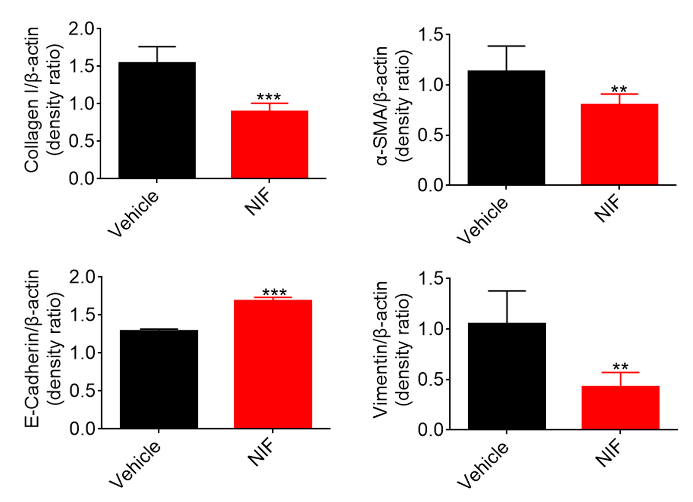
Figure S3 Bar graph showing the mean data from all subjects analyzed in each group in Fig 6e. Statistical significance was tested using Student’s t test; each point represents the mean ± SD for at least 3 independent experiments (**P < 0.01; ***P < 0.001 vs. vehicle control)


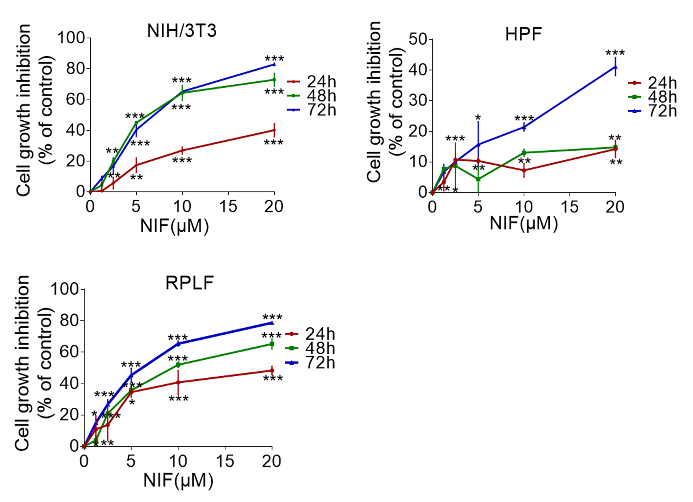


Figure S4 NIH/3T3, HPF, and rat primary lung fibroblasts (RPLF) were treated with diﬀerent concentrations of NIF for 24, 48 or 72 h and cell viability was measured by the MTT assay. Statistical significance was tested using Student’s t test; each point represents the mean ± SD for at least 3 independent experiments (*P < 0.05; **P < 0.01; ***P < 0.001, vs. Vehicle control)
